# Corrosion inhibition mechanism of a functionalized schiff base–derived quaternary ammonium salt for carbon steel in 1 M HCl: electrochemical, adsorption, and theoretical studies

**DOI:** 10.1038/s41598-026-41236-5

**Published:** 2026-04-04

**Authors:** Mohamed I. Ahmed, M. Abd-El-Raouf, M. A. Migahed, Magdy A.M. Ibrahim, Sameh A. Rizk, N. M. El Basiony

**Affiliations:** 1 Egyptian Petroleum Research Institute, Nasr City, Cairo, 11727 Egypt; 2 Chemistry department, Faculty of Science, Ain Shams University, Cairo, Egypt

**Keywords:** Carbon steel corrosion, Quaternary ammonium salt, Adsorption mechanism, Electrochemical impedance spectroscopy, Density functional theory, Monte Carlo simulation, Chemistry, Materials science

## Abstract

To address costly corrosion and material degradation, we report the development of a new quaternary ammonium salt (Q-Ar) via functionalization of a Schiff base with benzyl chloride through a quaternization reaction, intended as a potential corrosion inhibitor for carbon steel (C-steel) in 1 M HCl. FT-IR and ^1^H NMR spectroscopy confirmed the chemical structure of Q-Ar. Electrochemical potentiodynamic polarization (PDP) studies revealed that Q-Ar acted as mixed-type inhibitor and achieved corrosion inhibition efficiency 93.94% at 35 ppm. Electrochemical impedance spectroscopy (EIS) confirmed strong adsorption layer with charge transfer resistance (R_ct_) of 737.3 Ω.cm^2^. The adsorption behavior followed the Langmuir isotherm model, suggesting chemical adsorption reaction of Q-Ar at room temperature over C-steel surface according to standard adsorption energy values (∆G_ads_) of -43.2 kJ.mol^− 1^ for Q-Ar. Activation thermodynamic parameters of C-steel reaction in presence of Q-Ar suggested its inhibition action forming barrier film over C-steel surface as reflected by the increase in activation energy (Eₐ) from 21.60 kJ mol^− 1^ for the uninhibited system to 42.79 kJ mol^− 1^ in the presence of Q-Ar. Scanning electron microscopy (SEM) confirmed the protective effect of Q-Ar, showing that the C-steel surface remained largely free of corrosion products compared with the severely corroded blank sample. Energy-dispersive X-ray (EDX) analysis further verified this observation by revealing a marked reduction in iron oxide formation in the presence of Q-Ar. Theoretically, the lower energy gap (ΔE_gap_) of the optimized Q-Ar molecule and the higher adsorption energy (E_ads_) of the Q-Ar/Fe(110) system indicate strong interactions between the Q-Ar and the C-steel surface, corroborating the experimental observations.

**Supplementary Information:**

The online version contains supplementary material available at 10.1038/s41598-026-41236-5.

## Introduction

Corrosion in the petroleum field is a significant concern that can have detrimental effects on the integrity and performance of various equipment and infrastructure associated with the production, refining, and transportation of petroleum products^1^. Material corrosion refers to the degradation of a material, typically metal, due to chemical reactions with its surrounding environment, resulting in the loss of metal integrity and the deterioration of equipment^2,3^. C-steel is the essential material used in manufactory pipelines, tanks, heat exchanger, and drilling pipelines in petroleum production attributed to their low cost, high strength, and abundance^4–6^.So the damaged of the C-steel as a result of the corrosion should be replaced and/or repaired. One of the common industrial procedures used in the petroleum field that causes corrosion is acid cleaning which is aimed at eliminating scale and contaminants deposits on the surfaces of metallic equipment^7–9^.During the acid cleaning process, a diluted mineral acid, commonly HCl, is injected to dissolving scale and contaminants deposits^10,11^.So, the prevention of metal corrosion by acid cleaning solutions is essential. Corrosion prevention and control in the petroleum field involve a combination of material selection, coatings, cathodic protection, and corrosion inhibitors, in the acid cleaning process corrosion inhibitors were added to minimize the risk of corrosion^12^.Two factors are taken into account when selecting the inhibitor. First, it is easily synthesized from inexpensive raw ingredients. Second, The existence of an electron cloud, the electronegative atoms of sulfur, nitrogen, and oxygen, and the relatively long chain compounds cause increased adsorption on the metal surface to promote effective inhibition^13,14^. The architectures of many effective organic inhibitors include heteroatoms, multiple bonds, or aromatic rings. Quaternary ammonium salts are an example of these compounds and have been shown to be quite successful in preventing corrosion in a variety of harsh conditions^15–17^.Popova A. et al.^18^ investigated variant compounds of quaternary ammonium bromides of different heterocyclic compounds (TBB, T5T, and TP) as corrosion inhibitors for mild steel in 1 M HCl using polarization and gravimetric methods. Additionally, gravimetric measurements were conducted in 1 M H_2_SO_4_ for comparison. It was shown that the inhibitor’s molecular shape and concentration both affected its efficiency, TBB, T5T, and TP provide optimal protection. All compounds show a rise in inhibitor efficiency with concentration, as expected. The chemical structure of the molecule (ion) significantly impacts the inhibitory effects of the studied compounds. Increased surface area leads to improved inhibitor behavior. This series relies heavily on the ability of replacements to change conformation, resulting in increasingly condensed adsorbed layers. On the other hand, Hegazy M.A. et al.^18^ Investigated the impact of three novel di-quaternary ammonium salts (Q1, Q2, Q3) as corrosion inhibitors for *API X65* steel pipeline in 1 M HCl. The results showed that as the temperature was lowered and the concentration of inhibitor was increased, the inhibition efficiency values rose. The order of the rise in inhibitory efficiency was Q3 > Q2 > Q1.The compounds functioned as mixed-type inhibitors, blocking the active sites that produced the inhibition. Using inexpensive, commercially accessible precursors. Dheeraj S. C. et al.^19^, created two quaternary ammonium compounds (compound 2, compound 3) that their structures were confirmed by several spectroscopic techniques. These compounds were assessed utilizing surface analysis, electrochemical tests, and computational research corrosion inhibitors for C-steel surfaces in 1 M HCl solution. Both compound 2, compound 3 demonstrated high water solubility and were easily soluble in the 1 M HCl solution. The polarization resistance increased as the inhibitor dosage was increased, according to the EIS experiments, which validated the inhibitors adsorption on the metal substrate. Compound 3exhibited a superior adsorption behavior than compound 2, with an inhibitory efficiency of approximately 93%at a concentration of 200 mg L^− 1^.

The present study introduces a newly designed quaternary ammonium salt (Q-Ar) based on a Schiff base framework as an efficient corrosion inhibitor for carbon steel in 1 M HCl. Unlike many previously reported inhibitors, Q-Ar exhibits high interfacial adsorption affinity due to multiple electron-rich functional groups that enable strong donor–acceptor interactions with the steel surface. The inhibitor demonstrates remarkable efficiency at low concentration and maintains its protective performance under elevated temperatures and extended immersion times, closely resembling harsh industrial conditions. Q-Ar is highly soluble in aqueous acidic media, ensuring uniform adsorption on the carbon steel surface. Furthermore, being a Schiff base–derived quaternary ammonium compound, Q-Ar is generally considered low-toxicity and environmentally benign, although comprehensive ecotoxicity studies would be required for large-scale applications. The innovative combination of electrochemical techniques (EIS and PDP), surface analyses (SEM/EDX), and theoretical approaches (DFT and Monte Carlo simulations) demonstrates a strong correlation between molecular electronic properties, adsorption behavior, and inhibition performance, significantly advancing the understanding of structure–performance relationships for quaternary ammonium–based corrosion inhibitors.

## Experiments

### Synthesis of quaternary ammonium salt (*Q-Ar*)

**Scheme 1:**
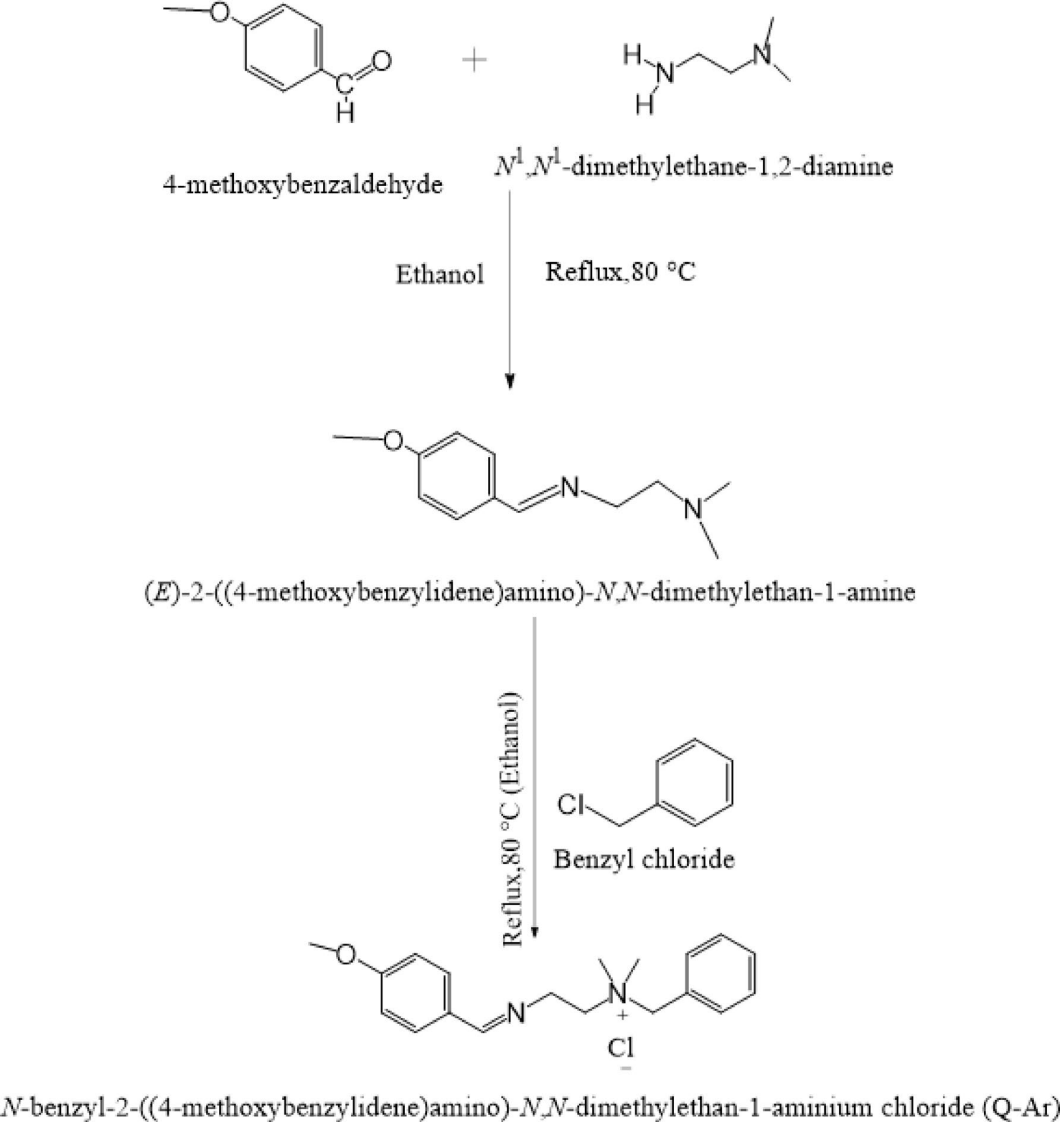
Synthesis way of quaternary ammonium salt (Q-Ar).

Quaternary ammonium salt (*Q-Ar*) was synthesized from the Schiff base according to the following steps (scheme1).

**Fig. 1:**
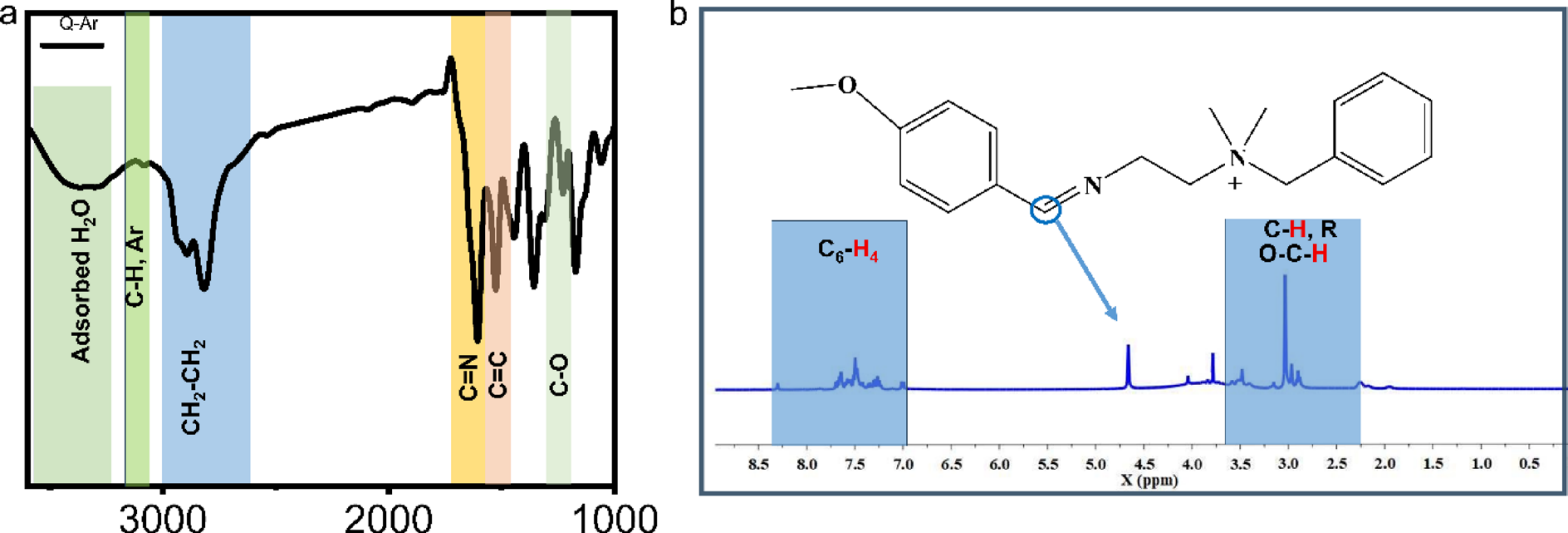
FTIR (**a**) and ^1^HNMR (**b**) spectra of Q-Ar.

Schiff base compound namely (E)−2-((4-methoxybenzylidene)amino)-N, N-dimethylethan-1-amine was prepared by reaction of 4-methoxybenzaldehyde (0.02 mol, 2.72gm) with N1,N1-dimethylethane-1,2-diamine (0.02 mol, 1.76gm) in molar ratio 1:1 in the presence of ethanol for 8 h. The reaction was cooled, purified using diethyl ether, and recrystallized with ethanol^20^. *Q-Ar* prepared through a quaternization reaction using the obtained Schiff base with Benzyl chloride for 72 h. The solvent was distilled off to give black crystals of *Q-Ar*^21^. The *Q-Ar* chemical structure was confirmed using *FTIR.* Figure 1a showed the *FTIR* absorption bands of Q-Ar at 3079 cm^− 1^ (C-H aromatic stretching), 2893 cm^− 1^ and 2817 cm^− 1^ (CH aliphatic), 1610 cm^− 1^(C = N) proves the occurrence of the condensation reaction, 1521 cm^− 1^(C = C aromatic), 1357 cm^− 1^(CH_3_), 1300 cm^− 1^(C–N), 1227 cm^− 1^(O–C). The broad band at 3367 cm^− 1^ can be explained by the vibration of adsorbed H_2_O owing to the hydroscopic nature of the quaternary salts. The chemical structure of *Q-Ar* was further validated by ^1^HNMR spectra as seen in Fig. 1b showing different characterized proton peaks for *Q-Ar* at 1.97 (s, 6 H, N^+^), 2.42 (t, 2 H, **N**^**+**^**CH**_**2**_CH_2_ *J =* 7.62 Hz), 2.97(s, 3 H, **OCH**_**3**_), 3.53 (t, 2 H, N = CH C**H**_**2**_, *J =* 7.62 Hz), 4.66 (s, 2 H, N^+^ **CH**_**2**_C_6_H_5_)), 7.01–7.55 (m, 4 H, **C**_**6**_ **H**_**4**_**)**, 7.60–7.85 (m, 5 H, **C**_**6**_ **H**_**5**_),) and 8.30 (s, 1H, N = **CH**).

**Solutions**.

Different concentrations of Q-Ar inhibitor (1–35 ppm) were prepared by serial dilution of a 1000 ppm stock solution in 1 M HCl at room temperature.

### Electrochemical techniques

A 100 mL double-jacketed cell was used for the electrochemical experiments, employing a three-electrode configuration. A *C-steel* electrode (1 cm²) of chemical composition *C-steel* ingredients in wt%; (C = (0.12 ~ 0.20), Si= (0.20 ~ 0.55), Mn= (1.20 ~ 1.60), P= (≤ 0.045), S= (≤ 0.045), Cr= (≤ 0.30), Ni= (≤ 0.30), Cu= (≤ 0.30) and Fe balanced served as the working electrode, with its potential recorded against the Ag/AgCl reference electrode, and the electrode of Pt wire acted as the counter electrode and connected together to a PGZ 402 potentiostat (Volta lab 80). After immersing the *C-steel* electrode for 30 min, this time frame is adequate to achieve a steady-state potential, the *EIS* and *PDP* measurements were conducted at varying concentrations of *Q-Ar*. *EIS* measurements were conducted over a frequency range of 100 kHz to 0.1 Hz with an amplitude of 10 mV. *PDP* measurements were performed at a scan rate of 1 mV/s, with the potential swept from − 800 to −200 mV.

### Surface analysis

After 6 h of immersion in 1 M HCl solutions containing either 35 ppm of the Q-Ar compound or no inhibitor (blank), the surface morphologies of the carbon steel sheets were examined using field-emission scanning electron microscopy (FE-SEM, JSM-IT800). The elemental composition of the outermost layer was analyzed by energy-dispersive X-ray spectroscopy (EDX).

### Quantum studies

Derived from DFT calculations, various quantum chemical parameters—such as the energies of the highest occupied molecular orbital (HOMO) and lowest unoccupied molecular orbital (LUMO), chemical hardness (η), energy gap (ΔE_gap_), and fraction of electrons transferred (ΔN)—were computed after optimizing the Q-Ar molecule. All calculations were performed using the DMol³ module within BIOVIA Materials Studio 6.0. The calculations employed the local density approximation (LDA) with the Perdew–Wang (PWC) correlation functional. The DMol³ electronic settings included the DNP(Double Numerical plus Polarization) basis set, a basis file of 3.5, and a medium orbital cutoff quality.Monte Carlo simulations (MCs) were performed using the Adsorption Locator module in the same software. The adsorption energy (E_ads_) for the interaction between Q-Ar and the Fe surface was calculated by importing an iron crystal cleaved along the (110) plane, which represents the most stable surface. The cleavage plane was expanded into a (10 × 10) supercell, and a 10 Å vacuum slab was added above the Fe(110) surface to eliminate periodic boundary effects. Simulated annealing was performed using 10 cycles with 15,000 steps per cycle which was sufficient to reach a stable minimum-energy configuration of the Q-Ar/Fe(110) system. The annealing process was controlled by the COMPASS force field to achieve the minimum-energy configuration of the Fe(110) surface. Electrostatic interactions were treated using the Ewald summation method, while van der Waals interactions were calculated using atom-based summation. The MC simulations were conducted in both vacuum and a simulated acidic environment consisting of 100 H_2_O molecules, 5 H_3_O^+^ ions, and 5 Cl^−^ ions^22^.

## Results and discussion

### OCP

**Fig. 2:**
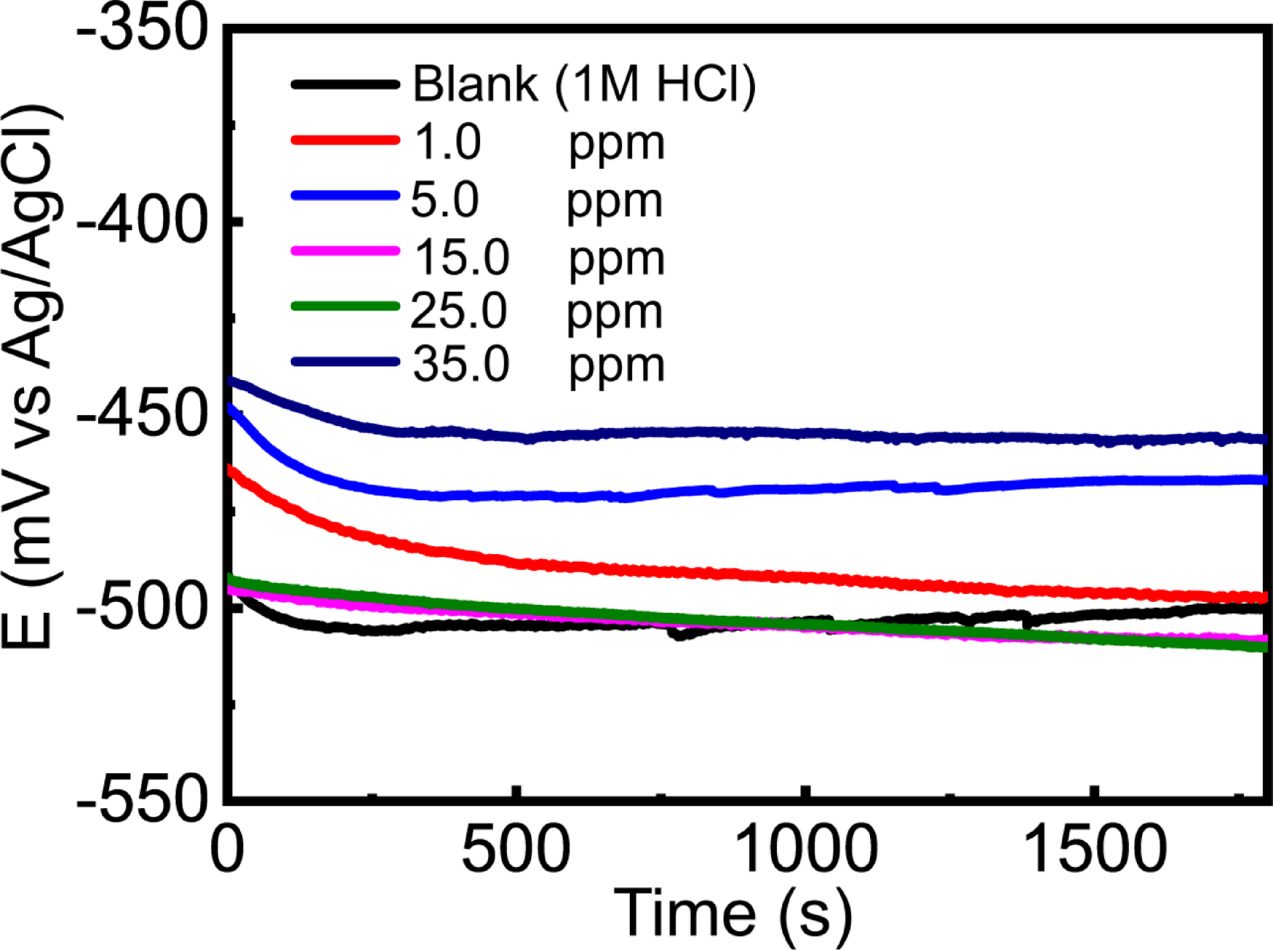
OCP curves of C-steel in 1 M HCl in presence and absence of different ppm doses of Q-Ar.

It is essential to allow the C-steel electrode to attain a stable open circuit potential (OCP) in 1 M HCl solution prior to conducting any electrochemical measurements, both in the absence and presence of different concentrations of the quaternary ammonium salt inhibitor (Q-Ar). Achieving OCP stabilization ensures that the metal/electrolyte interface reaches a quasi-equilibrium state, thereby providing reliable and reproducible electrochemical data. The evolution of the OCP of C-steel as a function of immersion time in uninhibited and inhibited acidic solutions is presented in Fig. 2. In the blank 1 M HCl solution, the OCP initially shifted toward more negative values before gradually stabilizing, which can be attributed to the rapid dissolution of the native oxide layer and the dominance of anodic iron dissolution accompanied by hydrogen evolution. The steady-state potential observed after prolonged immersion reflects the establishment of a dynamic balance between metal dissolution and corrosion product formation. In contrast, in the presence of Q-Ar at different concentrations, the OCP curves exhibited a noticeable shift toward more positive (noble) potentials compared to the blank solution. This positive displacement indicates a suppression of the anodic dissolution process due to the adsorption of Q-Ar molecules on the C-steel surface. As the inhibitor concentration increased, the OCP stabilization occurred more rapidly and at more positive potentials, suggesting enhanced surface coverage and stronger interaction between the inhibitor molecules and the metal surface. The stabilization of the OCP in inhibited solutions indicates the formation of a protective interfacial layer. In the uninhibited solution, this layer is mainly composed of porous and loosely adherent corrosion products, such as iron chlorides and iron oxides. However, in the presence of Q-Ar, the surface is covered by a smoother, denser, and more compact adsorbed organic film, which effectively isolates the metal surface from the aggressive acidic environment. This adsorbed film acts as a physical barrier that limits charge transfer and reduces the accessibility of corrosive species (H⁺ and Cl⁻) to the C-steel surface^23^.

**EIS**.

**Fig. 3:**
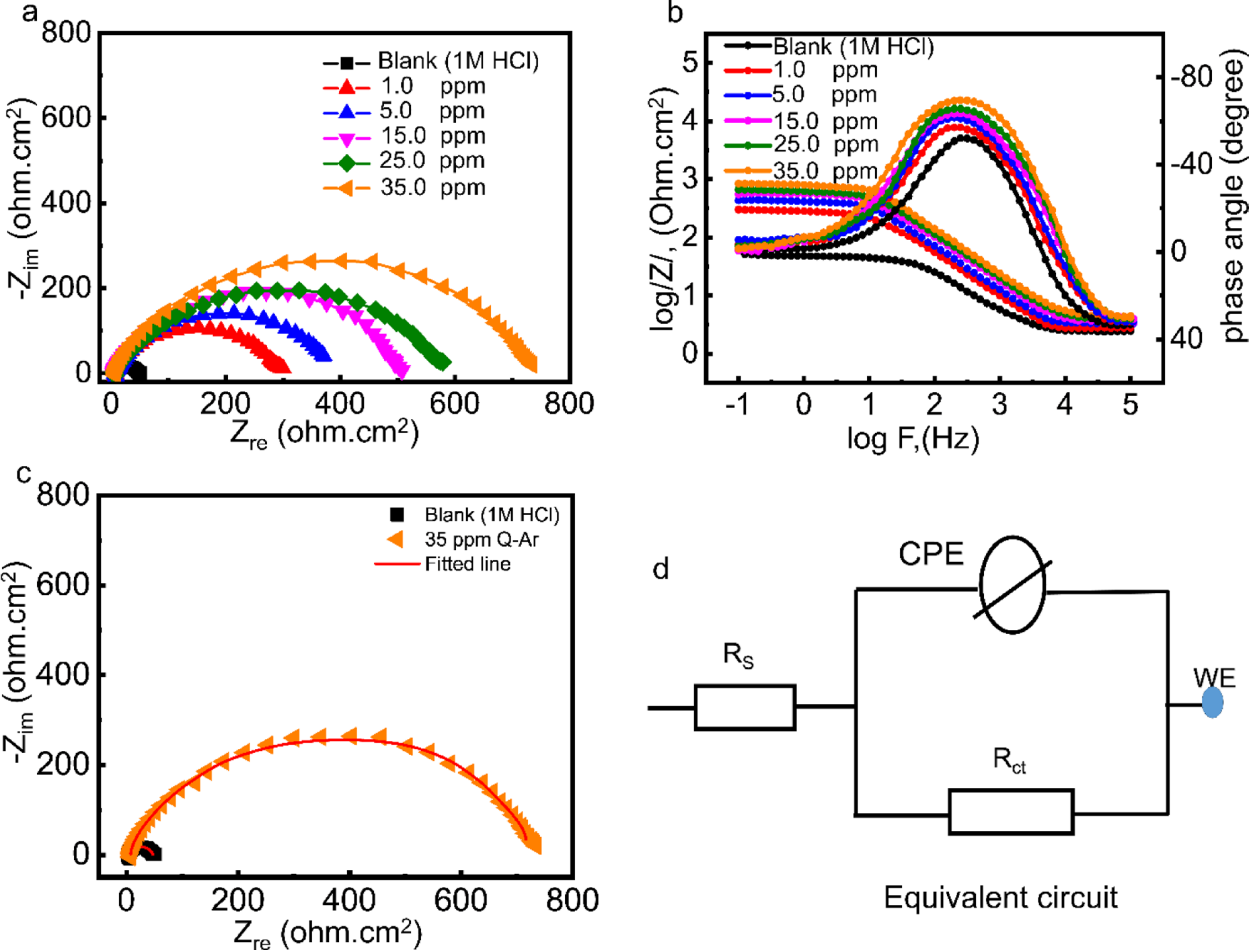
Nyquist (**a**) and Bode-phase angle plots (**b**) for C-steel in 1 M HCl in presence and absence of different concentration of Q-Ar, simulated Nyquist plot of C-steel in blank and after adding 35 ppm (c) using the proposed equivalent circuit (d).

EIS was employed to ascertain the influence of concentration and molecular structure of the *Q-Ar*, on the *C-steel* corrosion in 1 M HCl at ambient temperature. Figure 3**(a) and (b)** depict the Nyquist and Bode plots of *C-steel* in free HCl and after the addition of *Q-Ar*. The diameter of the Nyquist plots (Fig. 3**(a))** is directly connected to the charge transfer resistance (*R*_*ct*_) of the *C-steel*, which represents the resistance to the C-steel ionization process. The increase in Nyquist plot diameter with rising *Q-Ar* concentration indicates an enhancement in *R*_*ct*_, reflecting the improved corrosion inhibition provided by *Q-Ar*. This inhibition is attributed to the adsorption of *Q-Ar* molecule onto the C-steel surface^24^.This observation was confirmed by the increased separation between the Bode curves at lower frequencies and the shift of the phase angle curves towards − 90° in the intermediate frequency region, as seen in Fig. 3**(b)**^25–27^.The similar shapes of the Nyquist and phase-Bode curves indicate that the corrosion mechanism of C-steel remains unchanged and is controlled by a charge transfer process^28,29^.The Nyquist plots exhibited an imperfect semicircle shape, deviating from perfect circularity. This deviation can be attributed to frequency dispersion phenomena arising from relaxation processes related to charge transfer, diffusion, adsorption/desorption, and surface roughness characteristics of the C-steel^30,31^. The EIS results shown in Fig. 3d were analyzed using equivalent circuits comprising the solution resistance (R_s_ ​), charge-transfer resistance (R_ct_​), and the double-layer capacitance (C_dl_), which was replaced by a constant phase element (CPE) to improve the fit quality. The goodness of fit was evaluated using χ² values in the range of 10^− 3^–10^− 4^, confirming excellent agreement between the experimental and simulated data, as illustrated in Fig. 3c. Based on *R*_ct_ values of C-steel in presence of Q-compound (*R*_ct.Q_) and their absence (*R*_ct. HCl_), surface coverage (*θ*_*z*_) and the inhibition efficiency (*IE*_*z*_*%*) were calculated using the corresponding equations:

1 $$\:\theta\:=({R}_{ct.Q}-{R}_{ct.HCl})/{R}_{ct.Q})$$

2 $$\:{IE}_{Z}\%=\:{\theta\:}_{\mathrm{z}}\:x100$$

The *EIS* parameters are summarized in Table 1. The presence of *Q-Ar* significantly increased the *R*_*ct*_ due to their adsorption onto the *C-steel* surface. This adsorption process effectively lowered the *C-stee*l corrosion rate by replacing adsorbed water molecules and chloride ions on the metal surface with *Q-Ar*. As the concentration of *Q-Ar* increased, *R*_*ct*_ values rose, reaching 737.3 Ω.cm^2^ at 35 ppm. This increase in *R*_*ct*_ indicates that the presence of *Q-Ar* enhanced the resistance of *C-steel* to the corrosion process. The increase in *Q-Ar* concentration reduces the effective surface area of *C-steel* available for charge transfer. This reduction leads to a decrease in capacitance, as described by the Helmholtz Eq. (3).

3 $$\:{C}_{\mathrm{d}\mathrm{l}}=\left(\frac{{\epsilon\:}^{^\circ\:}\epsilon\:}{T}\right)A$$

Where, *A* is electrode surface area, *ε₀* is permittivity of free space, *ε* is local dielectric constant and *T* is thickness of the adsorbed layer.

The formation of Q-Ar film on the *C-steel* surface, replacing adsorbed water molecules, increases the thickness (T) of the adsorbed layer. This increased thickness contributes to the observed decrease in capacitance^32,33^. The EIS data in Table 1 show that the nnn-values of the constant phase element (CPE) slightly decrease from 0.864 for the blank to a range of 0.801–0.843 in the presence of Q-Ar, indicating minor surface heterogeneity caused by adsorption of the inhibitor^34^. The relaxation time (τ), calculated as the product of the charge-transfer resistance (R_ct_​) and the double-layer capacitance (C_dl_​), provides insight into the dynamics of the electrochemical interface. As shown in Table 1, τ increases from 2.65 × 10^− 3^ s for the blank C-steel to values between 1.02 × 10^− 2^ s and 1.29 × 10^− 2^ s in the presence of Q-Ar, reaching a plateau at higher concentrations. This increase indicates that the charge-transfer process slows due to the formation of a compact and protective adsorption layer of Q-Ar molecules on the C-steel surface^35^. The calculated surface coverage (θ) and inhibition efficiency (IE_z_ %) increase progressively with increasing inhibitor concentration, confirming that Q-Ar effectively blocks both anodic and cathodic active sites. The inhibition efficiency reaches a maximum of 93.73% at 35 ppm. The adsorption of Q-Ar onto the carbon steel surface occurs primarily through its active centers, including nitrogen and oxygen atoms, as well as the π-electrons of C = N bonds and aromatic rings. The excellent inhibition performance of Q-Ar can be attributed to its molecular structure, which features multiple benzene rings that enhance planarity and promote strong donor–acceptor interactions with the metal surface. These interactions facilitate the formation of a compact and protective adsorption layer, resulting in significantly improved corrosion inhibition efficiency^36^.

**Table 1:** *EIS* parameters for *C-steel* 1 M HCl in absence and presence of different concentrations of *Q-Ar* at room temperature.

Inh.	Conc, ppm	*R*_s_, (Ω.cm^2^)	*R*_ct_, (Ω.cm^2^)		C_dl_, (µF/cm^2^)		Ɵ_z_	IE_z_%
				*n*		,sτ		
**Blank**	0.00	3.16	46.2	0. 864	57.344	0.0026	---	---
***Q-Ar***	1	3.28	295.2	0.831	34.41	0.0102	0.8434	84.34
	5	3.68	385.8	0.843	31.05	0.0120	0.8802	88.02
	15	3.83	502.7	0.841	25.26	0.0127	0.9080	90.80
	25	3.93	589.8	0.801	21.845	0.0129	0.9216	92.16
	35	3.79	737.3	0.807	17.278	0.0127	0.9373	93.73

### PDP

**Fig. 4:**
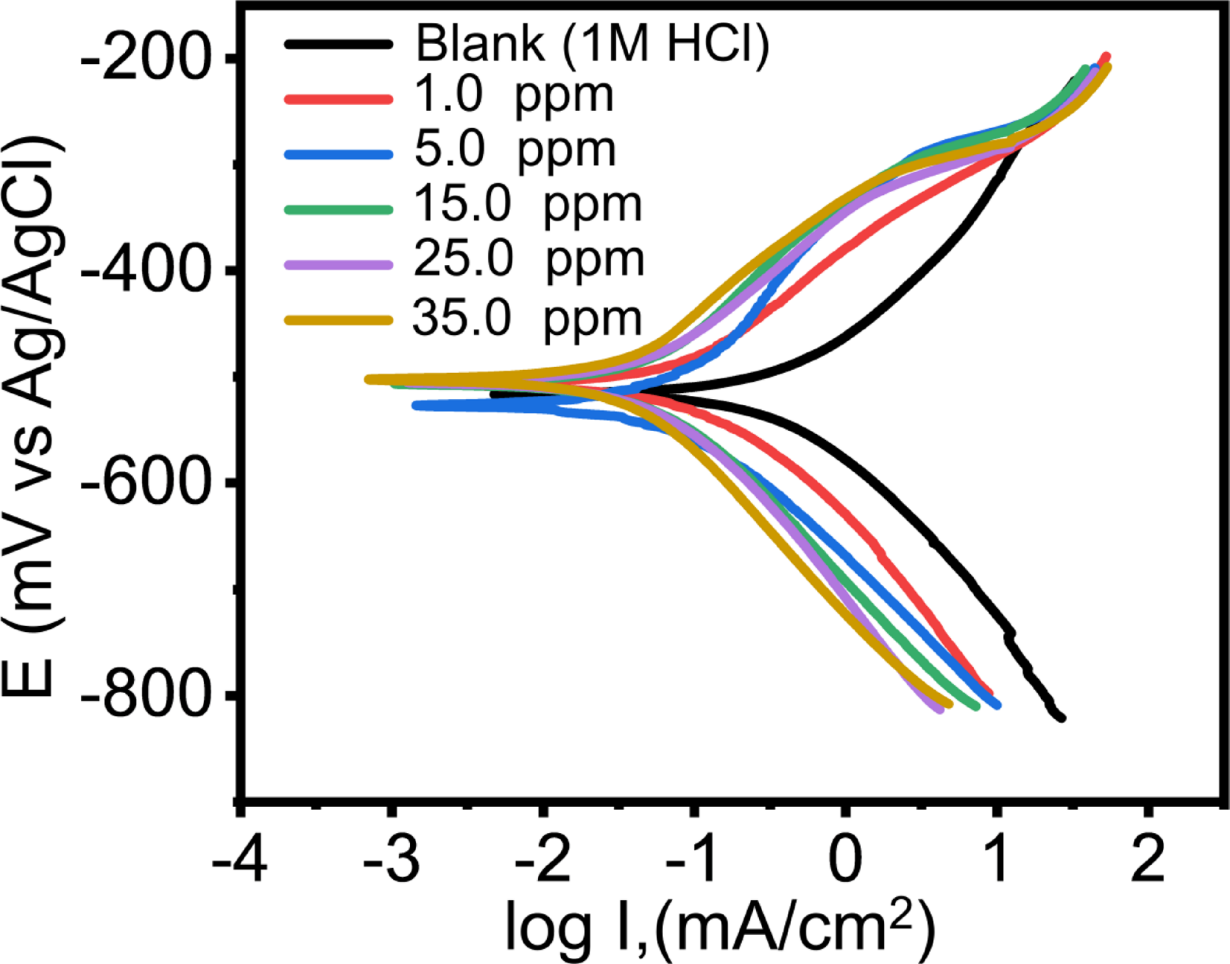
Potentiodynamic polarization (PDP) curves of C-steel in 1 M HCl in the absence and presence of different concentrations of Q-Ar.

Figure 4 demonstrates the polarization curves of *C-steel* in 1 M HCl solution in the absence and presence of *Q-Ar* inhibitor at room temperature. The addition of *Q-Ar* resulted in a shift of the Tafel curves towards lower current densities. This shift indicates the inhibition effect of this compound, which is due to the fact that its adsorption on the *C-steel* surface. Also, the adsorption of *Q-Ar* increases with their concentration due to the accumulation effect of Q-Ar molecules on the C-steel^37^. Figure 4Q*-Ar* exhibited a clear anodic inhibition effect within lower sweeping potential regions. However, at higher anodic potentials (> −330 mV), the inhibition performance of Q-Ar inhibitor diminished. This potential, known as the desorption potential, indicates that the desorption rate of the Q-Ar becomes more prominent than its adsorption at higher anodic potentials^38^. Consequently, the inhibition mode of *Q-Ar* is influenced by both its concentration and the applied potential. The parallel nature of the cathodic Tafel lines suggests that *Q-Ar* also inhibited the cathodic hydrogen evolution reaction (2$\:{H}^{+}+2{e}^{-}\to\:{H}_{2\uparrow\:})$. This finding confirms the adsorption of *Q-Ar* molecules on the surface of *C-steel* diminishes the interaction between H^+^ ions and the metal surface. Therefore, *Q-Ar* can inhibit both anodic and cathodic reactions on *C-steel*^39,40^. Furthermore, the similar shapes of the polarization curves for C-steel in the absence and presence of Q-Ar (Fig. 4) indicate that the fundamental corrosion reaction mechanism of C-steel remains unchanged^12,38^.

To gain insights into the corrosion kinetics, several electrochemical parameters were extracted from the polarization curves, encompassing the anodic and cathodic Tafel slopes (*βa*,* βc*), corrosion potential (*E*_*corr*_), along with corrosion current density (*i*_*HCl/Q*_). These values were determined by extrapolating the linear Tafel regions of the curves and are presented in Table 2.

**Table 2:** Tafel parameters for *C-steel* in absence and presence of different concentrations of *Q-Ar* at room temperature.

Inh.	Conc, ppm	E_corr_, (mV) vs. Ag/AgCl	i_corr_ (mA/cm²)	β_a_ (mV/dec)	-β_c_ (mV/dec)	CR (mm/year)	θ_*P*_	IE_*P*_%
**Blank**	0.00	−516.3	0.5712	120.7	133.8	6.626385	-----	-----
***Q-Ar***	1	−507.7	0.1207	138.3	173.1	1.400218	0.7886	78.86
	5	−526.8	0.0989	153.5	141.0	1.14732	0.8268	82.68
	15	−506.4	0.0783	143.5	156.7	0.908344	0.8629	86.29
	25	−505.0	0.0589	165.4	177.8	0.683288	0.8968	89.68
	35	−502.6	0.0346	130.5	142.9	0.401388	0.9394	93.94

The surface coverage (*θ*_*P*_), inhibition efficiency (*IE*_*p*_*%*) and corrosion rate (CR) were calculated using the following equations^34^:

4 $$\:\theta\:=({\mathrm{i}}_{\mathrm{H}\mathrm{C}\mathrm{l}}-{\mathrm{i}}_{\mathrm{Q}})/{i}_{\mathrm{H}\mathrm{C}\mathrm{l}}$$

5 $$\:IEp\%=\:{\theta\:}_{\mathrm{P}}\:X100$$

6 $$\:CR=\:0.00327\:x\:eq.wt\:x\:{i}_{\mathrm{c}\mathrm{o}\mathrm{r}\mathrm{r}}\:/{Fe}_{d}$$

where i_HCl_ and i_Q_​ are the corrosion current densities of C-steel in uninhibited 1 M HCl solution and in the presence of Q-Ar, respectively. The term *eq.wt* denotes the equivalent weight of iron (27.92 g equiv^− 1^), i_corr_​ is the corrosion current density expressed in µA cm^− 2^, and Fe_d_ is the density of iron (7.86 g cm^− 3^).

The addition of *Q-Ar* resulted in increased values of both the anodic Tafel slope (*βa*) and cathodic Tafel slope (*βc*). This indicates a decrease in the rate of both H_2_ evolution (cathodic) and iron dissolution (anodic) reactions^41,42^. The observed slight variation in corrosion potential (*E*_*corr*_) values (less than 85 mV) suggests a mixed-type inhibition mechanism, where *Q-Ar* blocks both cathodic and anodic sites^43–45^. The inhibition is likely explained by the development of an adsorbed film of *Q-Ar* on the C-steel surface, facilitated by the interaction between the 3d-orbitals of Fe and the active centers of the *Q-Ar*. These active centers include heteroatoms (O and N), N = C double bonds, benzene rings, and the positive charges present in the *Q-Ar*. The corrosion current density (*i*_*corr*_) decreased with increasing inhibitor concentration, reaching values of 32.9 µAcm^− 2^ at the optimum concentration (35 ppm) for *Q-Ar*. This reduction in i_corr_ corresponds to a significant decrease in the corrosion rate of C-steel, confirming the high inhibitive performance of the synthesized quaternary ammonium salt due to its strong adsorption affinity toward the Fe surface^46,47^. Furthermore, the inhibition efficiency (*IE*_*p*_%) increased with increasing inhibitor concentration, reaching 93.94% at 35 ppm. This can be attributed to the increased surface coverage achieved by the adsorbed *Q-Ar* molecules, which effectively shield the *C-steel* surface against the corrosive environment^48,49^. Table 3S highlights the superior performance of the investigated Q-Ar compared to recently reported corrosion inhibitors under similar experimental conditions.

### Thermodynamic parameters

The influence of temperature (293, 303, 318, and 333 K) on the PDP of *C-steel* in 1 M HCl solutions was investigated in the absence (Fig. 1s **a**) and presence of 35 ppm *Q-Ar* (Fig. 1s **b**). As the temperature increased, the polarization curves shifted towards more active regions, indicating an accelerated dissolution rate of *C-steel*. This increased dissolution rate is accompanied by the evolution of more hydrogen gas, which can contribute to the desorption of corrosion products and the adsorbed inhibitor (*Q-Ar*) as well. Despite the detrimental effect of temperature, *Q-Ar* exhibited a relatively small loss in inhibition performance, decreasing by only 11.8%. This demonstrates that the Q-Ar has a significant potential for corrosion protection of C-steel in harsh environments^50–52^(Table 1s).

Based on the *C-steel* corrosion rate(*CR*) values, the activation energy (*E*_*a*_), activation entropy(ΔS*), and activation enthalpy (ΔH*) were calculated using Eqs. (6 and 7)^53^:

7 $$\:{Ln}CR\:=Ln\:A\:-(\frac{{E}_{a}}{RT})\:$$

8 $$\:Ln\left(\frac{CR}{T}\right)=\left[Ln\left(\frac{R}{h\:{N}_{A}}\right)+\left(\frac{{\varDelta\:S}^{*}}{R}\right)\right]-\left(\frac{{\varDelta\:H}^{*}}{RT}\right)$$

**Fig. 5:**
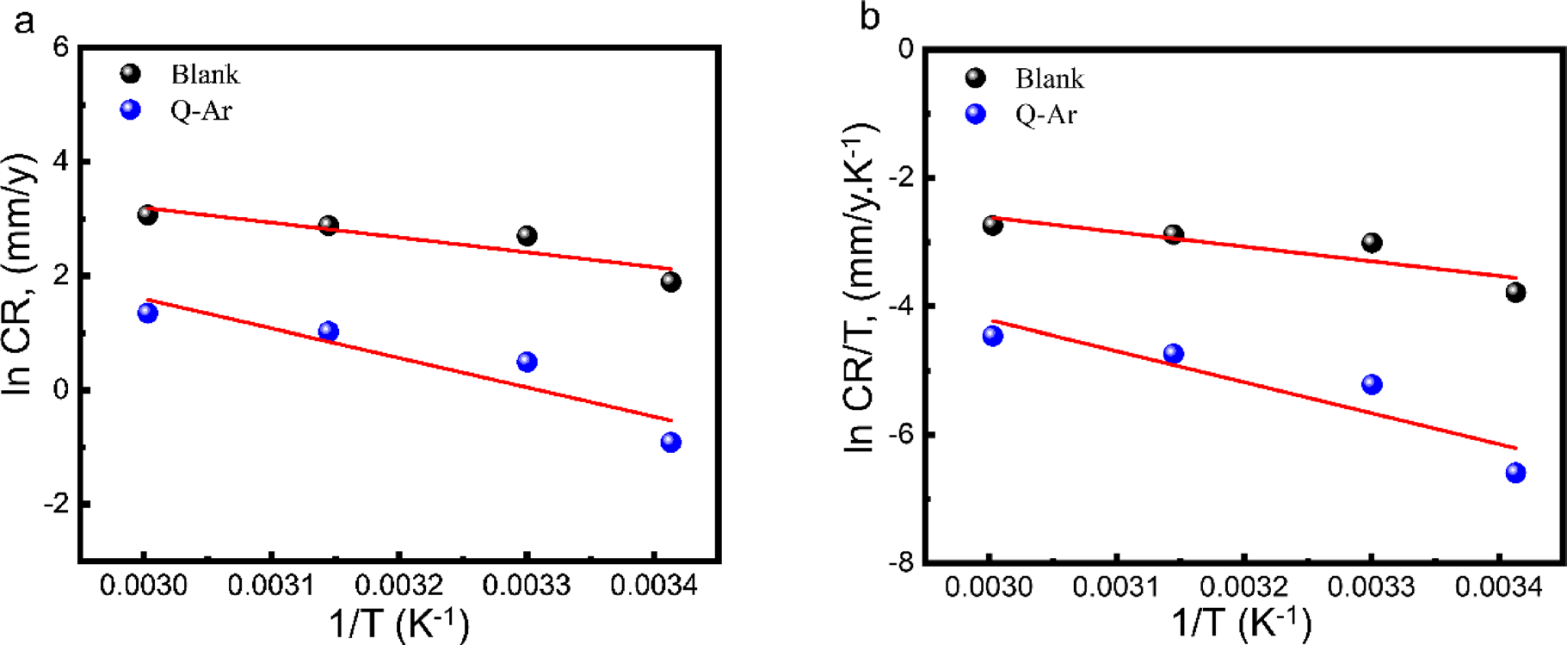
Arrhenius (a) and transition state (b) plots for C-steel in the absence and presence of 35 ppm of Q-Ar.

Where *A* symbolized Arrhenius constant. *N*_*A*_, *h*,* T*,and *R* are Avogadro’s number, Blank constant, absolute temperature, and gas constant, respectively. The slope of the Arrhenius plot (Fig. 5a), -Ea/8.314, is a function of the activation energy (*E*_*a*_) values as in Table 3 for the corrosion of *C-steel* in free HCl and in solutions containing 35 ppm *Q-Ar*. The increase in *E*_*a*_ values in the presence of *Q-Ar* suggests that this inhibitor introduce an energy barrier, hindering the corrosion process and reducing the corrosion rate of *C-steel*. This observation supports the notion of Q-Ar’s physical (electrostatic) adsorption onto the *C-steel* surface^54,55^.Additionally, the positive values of ΔH* (activation enthalpy, slope of Fig. 5b) increase in the presence of *Q-Ar*, reflecting the endothermic nature of C-steel corrosion reaction. This indicates that the dissolution of *C-steel* becomes more difficult or slower in the presence of Q-Ar^56^. The difference between the activation energy and the enthalpy of activation (E_a_ – ΔH* ≈ 2.6 kJ mol⁻¹) is close to RT, in agreement with transition state theory. This consistency confirms the validity of the kinetic analysis and agrees well with previously reported corrosion inhibition studies^57^. The more negative value of ΔS* (activation of entropy, intercept of Fig. 5b) for the free acid solution (−171.65 JK^−1^mol^− 1^) suggests that the rate-determining step involves the formation of a highly ordered activated complex (recombination). In contrast, the shift of ΔS* towards less negative values (−105.04 JK^−1^mol^− 1^) in the presence of *Q-Ar* indicates a decrease in the degree of order in the activated complex (Q-Ar–Fe). The presence of *Q-Ar* molecules on the C-steel surface can almost completely cover the surface, hindering the discharge process and leading to a more disordered system^58,59^. The calculated activation parameters (*E*_*a*_, ΔH*, and ΔS*) confirm that Q-Ar provides strong corrosion protection. This effectiveness is attributed to its molecular structure, which contains multiple electrostatic interaction centers such as the quaternary nitrogen atom, protonated methoxy group, and chloride ions. Additionally, the rigid π-electron system of Q-Ar provides several active sites for electron donation and possible chemisorptive interactions, resulting in the formation of a stable and protective adsorbed layer on the carbon steel surface.

**Table 3:** Activation thermodynamic parameters of *C-steel* in absence and presence of *Q-Ar*.

Inh.	slope		E_a_ (kJ mol^− 1^)	slope	intercept	ΔH* (kJ mol^− 1^)	-ΔS* (J mol^− 1^)
**Blank**	−2598.37		21.602	−2286.18	4.2435	19.007	162.257
***Q-Ar***	−5147.14		42.793	−4834.95	10.29205	40.197	111.969

### Adsorption isotherm

**Fig. 6:**
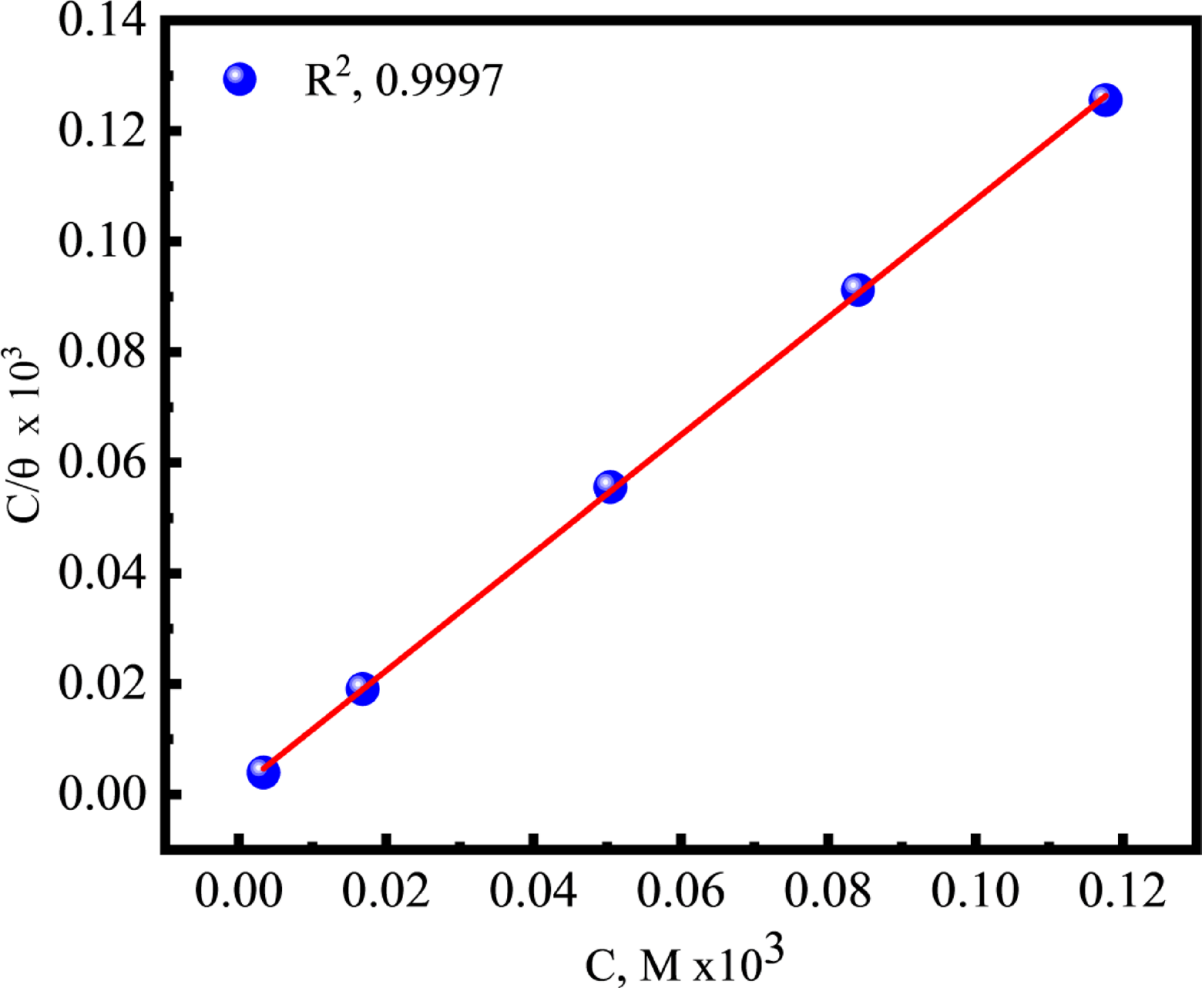
Langmuir adsorption isotherm of Q-Ar at C-steel/HCl interface at room temperature.

The adsorption of *Q-Ar* and its interaction with *C-steel* surface studied using various adsorption isotherms. Of the applied isotherm models and based on the EIS data, the Langmuir adsorption isotherm was the more fitted adsorption model to discuss the adsorption phenomena of *Q-Ar*, due to the higher correlation coefficient (*R*^*2*^) and the slope of Fig. 6 near the unity^60–62^.The standard free energy change (*ΔG*_*ads*_) of *C-steel* in the presence of *Q-Ar* at room temperature is a function of *K*_ads_ (equilibrium adsorption constant) according to the equation^63^:

9 $$\:{-\varDelta\:G}_{ads}=2.303RTlog\left(55.5x{K}_{ads}\right)$$

where (55.5) is the molar concentration of water and (*R*) is the standard gas constant. The greater *K*_*ads*_ value in Table 4, can be attributed to the high adsorption capability of *Q-Ar* on the surface of *C-steel*. This phenomenon is elucidated by the donor-acceptor interplay among the active centers in *Q-Ar* structure and the partially filled 3d-orbital of iron leading to the formation of a strong chemical bond^64^ The obtained negative values of $\:\varDelta\:{G}_{ads}$ for *Q-Ar* indicate the spontaneous nature of its adsorption process over *C-steel*. Furthermore, these values as in Table 4 signify the strong interaction and stability of the adsorbed layer with the *C-steel* surface. *ΔG*_*ads*_ was − 43.2 kJ.mol^− 1^ for *Q-Ar*, this indicated that *Q-Ar* adsorbed on *C-steel* surface chemically at room temperature, which attributed to the presence of activated centers like: quaternary-N, π-electrons (C = N and benzene ring), and hetero atoms (Nitrogen and oxygen atoms).

**Table 4:** Langmuir adsorption isotherm parameters of *C-steel* in absence and presence of *Q-Ar*.

EIS											
***Inh***.	***slope***			**R** ^**2**^		**K**_**ads**_, (L.mol^−1^)x10^5^		-∆G_ads_ (kJmol^−1^)			
*Q-Ar*		1.0639			0.9997		8.89		43.2		

### Effect of immersion time of the adsorbed film stability

**Fig. 7:**
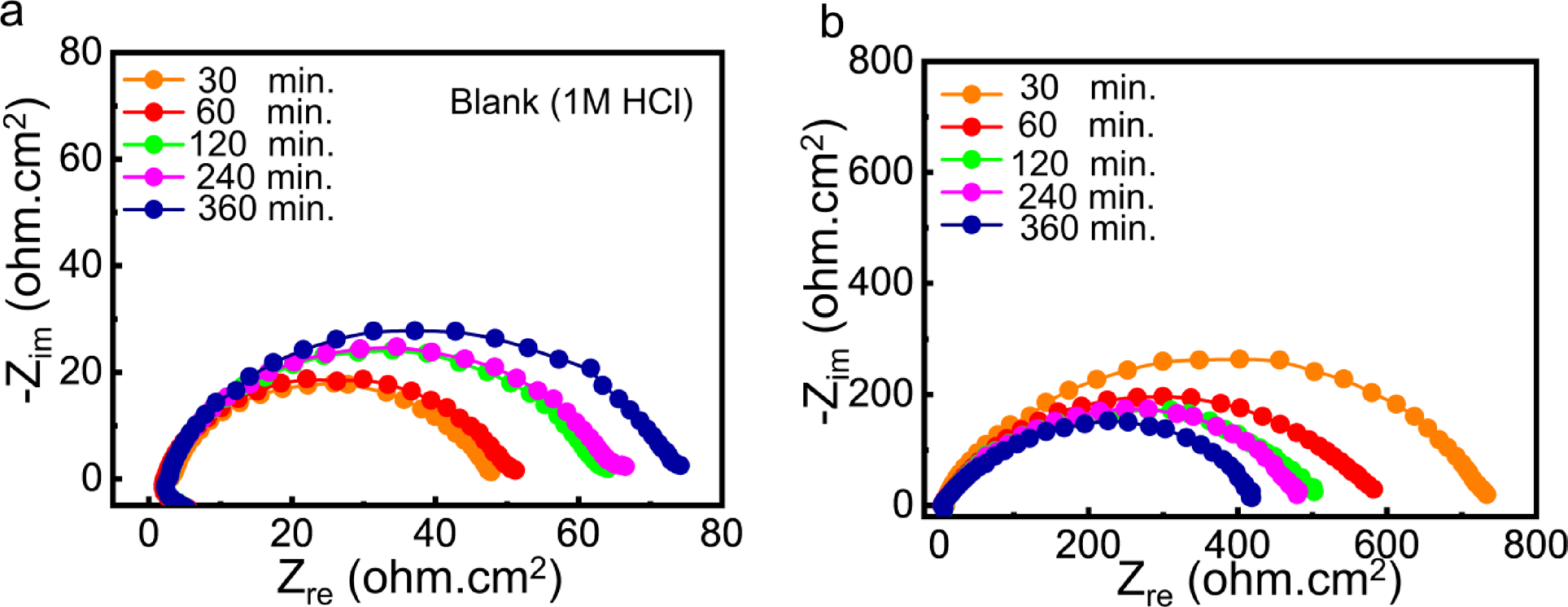
Nyquist plots of C-steel in free HCl (a) and after treatment with 35 ppm of Q-Ar (b) at different immersion time.

To evaluate the stability of the adsorbed Q-Ar film over time, Nyquist plots were recorded for C-steel in pure 1 M HCl (Fig. 7a) and in the presence of 35 ppm Q-Ar (Fig. 7b) at different immersion times. The similar shapes of the Nyquist plots at all immersion periods indicate that the corrosion mechanism of C-steel remains unchanged, confirming that the inhibition process is governed primarily by surface coverage rather than a change in reaction pathway. As summarized in Table 2S, the charge-transfer resistance (*R*_*ct*_) of C-steel in uninhibited HCl gradually increases to 74.08 Ω·cm² with immersion time, which can be attributed to the accumulation of corrosion products on the metal surface. In contrast, for the inhibited system, *R*_*ct*_ ​ decreases from its initial value to 466.2 Ω·cm² after prolonged immersion. This decrease is associated with partial desorption of Q-Ar molecules caused by continuous hydrogen evolution during immersion, which may locally disrupt the protective layer and slightly reduce its thickness^32,65^. Despite this decrease in R_ct_ ​, the inhibition efficiency remains high (83.12% after 6 h of immersion), indicating that the majority of the Q-Ar molecules remain strongly adsorbed on the C-steel surface. This behavior demonstrates the formation of a relatively stable and adherent adsorption film, capable of maintaining significant corrosion protection even under extended exposure conditions. The sustained inhibition performance can be attributed to the molecular structure of Q-Ar, which contains multiple adsorption centers and aromatic rings that enhance intermolecular interactions and promote strong binding with the steel surface^66,67^. The unique chemical structure of Q-Ar endows it with significant potential for application under prolonged immersion conditions.

### Quantum

#### Density function theory (DFT)

**Fig. 8:**
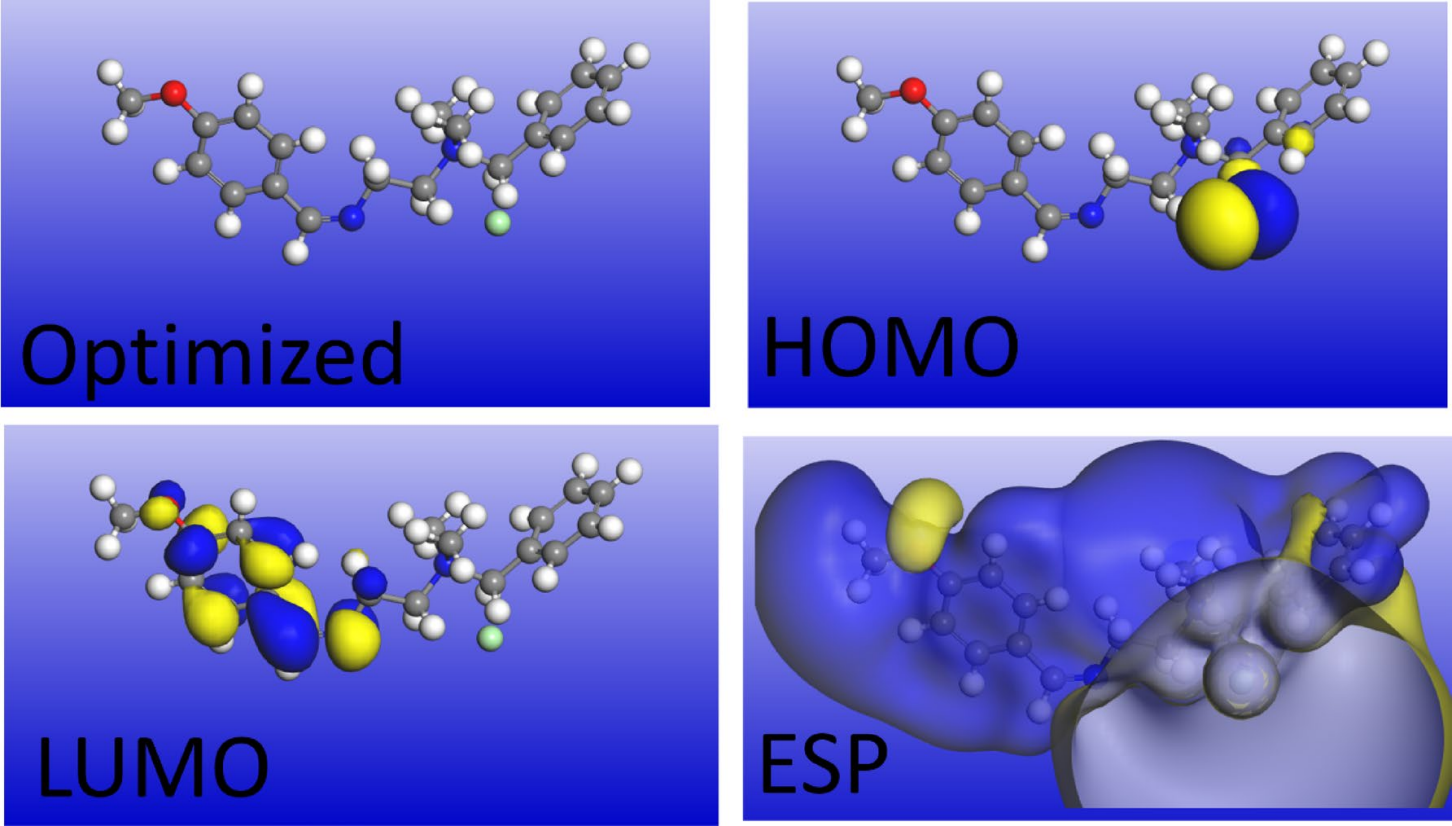
Optimized geometry structure, HOMO, LUMO, and ESP of the Q-Ar in neutral form.

*DFT* calculations applied to explore the correlation between the chemical structure of *Q-Ar* its adsorption affinity on the *C-steel* surface. Based on frontier molecular orbital (*FMO*) theory, the distribution of electron density over specific functional groups in the *Q-Ar* molecules was analyzed. Figure 8 presents the most stable configuration obtained from *DFT* calculations, including the optimized structure, molecular orbitals of the highest occupied (*HOMO*) and lowest unoccupied (*LUMO*) and electrostatic potential mapping (ESP) of the neutral form of Q-Ar while those of protonated from are displayed in Fig. 2Si. These *HOMO* and *LUMO* orbitals correspond to the nucleophilic and electrophilic centers, respectively. The optimized structure of *Q-Ar* exhibit a planar geometry, which enhances the accessibility of its active centers to the *C-steel* surface and ultimately improves its adsorption performance^33,68^.The electron cloud distribution in the *HOMO*, as visualized in the optimized structures is located on the benzene ring, quaternary nitrogen atoms and chloride ions (nucleophilic centers). This enhances the electrostatic attraction between the Q-Ar and the negatively charged *C-steel* surface, reducing the distance between them and enhance the π-electron interaction with the substrat^47^.Conversely, the *LUMO* orbitals are primarily located on the aromatic benzene rings (electrophilic centers), facilitating electron donation and back-donation processes from the 3d-orbital of Fe and anti-bonding orbital (π*) of Q-Ar. This increased electron interaction leads to the formation of stable *Q-Ar-Fe* complexe, which act as a shielding layer on the *C-steel* surface^47,69^. These findings are consistent with the PDOS results, which reveal significant overlap between Fe 3 d states and the inhibitor p/π states near the Fermi level, confirming the formation of stable Q-Ar–Fe chemical bonds. The resulting adsorbed complex forms an effective shielding layer that protects the C-steel surface from corrosive attack. Furthermore, the electrostatic potential mapping (ESP) analysis confirmes the FMO discussion, where the electron distribution over the Q-Ar enhances its effective adsorption.

Table 5 shows the output quantum indices derived using the following formula:

10 $$\:\varDelta\:{E}_{gap}={E}_{LUMO}-{E}_{HOMO}$$

11 $$\:\eta\:=\frac{\varDelta\:{E}_{gap}}{2}$$

12 $$\:\chi\:=\frac{-({E}_{HOMO}+{E}_{LUMO})}{2}$$

13 $$\:\varDelta\:N=\frac{({\phi\:}_{Fe}-{\chi\:}_{Q-compd.})}{\left[2\left({\eta\:}_{Fe}+{\eta\:}_{Q-compd.}\right)\right]}$$

14 $$\:{E}_{b-donation}=-\left(\frac{\eta}{4}\right)$$

Where, Δ*E*_*gap*_, *η*,* ΔN*,* χ*, and $\:\phi\:$ are energy gap, global hardness, fraction of transferred electron, electronegativity and work function of Fe (1 1 0) plan is 4.82eV^65,66^.

The data presented in Table 5 indicate that *Q-Ar* has a higher propensity to donate and accept electrons to or/from the 3 d orbitals of iron. This is supported by the higher energy values of the *HOMO* and the back-donation of *Q-Ar*. These findings suggest that *Q-Ar* exhibits superior donor-acceptor interaction performance with the *C-steel* surface, aligning with the observed experimental data^12,67^. Consequently, this highlights the potential of these compounds as effective corrosion inhibitors. *∆E*_*gap*_ is another significant quantum parameter that indicates the reactivity of Q-Ar. The smaller the *E*_*gap*_ value, the greater the electron polarizability, and the lower the hardness, the smallest $\:{E}_{gap}$ value of *Q-Ar* shows greater polarizability and reactivity features, which translate into stronger adsorption affinity or electron donation^70–72^. Lukovits’s results regarding the fraction of electron transference (∆*N* = 2.2) indicate that the material’s adsorption probability features increased as the *∆N* value grew^23^. To identify the most probable adsorption form of the investigated inhibitor on the C-steel surface, both neutral and protonated species were examined. Protonation sites were selected based on Mulliken atomic charge analysis, which allows identification of the most electron-rich atoms susceptible to protonation. According to the charge distribution, the oxygen atom O(9) in the Q-Ar molecule, carrying the highest negative charge (−0.465), was identified as the preferred protonation site. The electronic properties of the protonated structure were calculated using the same DMol³ computational parameters applied to the neutral form to ensure consistency. The optimized geometries and frontier molecular orbitals (HOMO and LUMO) of the protonated species are illustrated in Fig. 2S. A comparative analysis of the quantum chemical parameters for the neutral and protonated forms (Table 5; Fig. 2S) reveals that the protonated species exhibit higher HOMO energy values than their neutral counterparts. This increase in HOMO energy indicates an enhanced ability of the protonated form to donate electrons to the metal surface. Furthermore, the protonated species exhibit greater chemical reactivity, as indicated by their lower energy gap (ΔE) and higher fraction of electrons transferred (ΔN), which facilitates electron donation to the metal surface and enhances metal–inhibitor interactions. These observations suggest that the protonated form has a stronger adsorption affinity toward the C-steel surface compared to the neutral species^73^. Therefore, it can be concluded that protonated structures are more likely to dominate the adsorption process, playing a key role in the corrosion inhibition performance of Q-Ar in acidic media.

**Table 5:** Quantum chemical parameters of the investigated *Q-Ar compound* in neutral and protonated forms.

Quantum parameters	Q-Ar	Q-AR-H^+^
***E***_**H**_ **(eV)**	−4.123	−3.767
***E***_**L**_ **(eV)**	−2.44	−2.109
**∆** ***E*** **(eV)**	1.683	1.657
***X*** **(eV.mol**^**− 1**^**)**	3.2815	2.938
***η*** **(eV. mol**^**− 1**^**)**	0.8415	0.829
$\:{\boldsymbol{E}}_{\mathbf{b}\to\:\mathbf{d}}\:$ **(eV. mol** ^**− 1**^ **)**	0.2103	0.2072
$\:\varDelta\:\boldsymbol{N}$	2.209	2.451

#### Monte Carlo simulation (*MCs*)

**Fig. 9:**
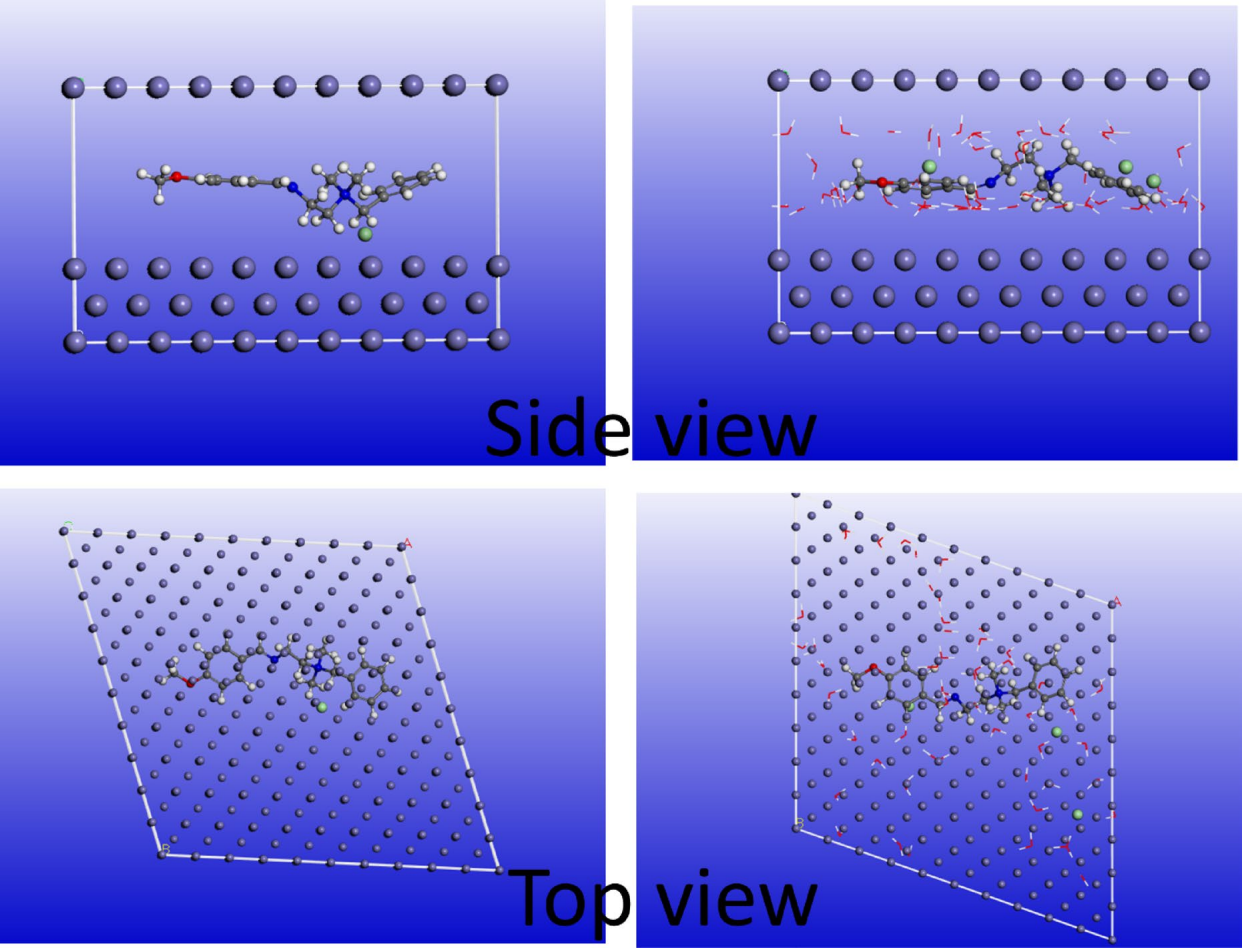
Side and top views of the adsorbed Q-Ar in gas and liquid phases on Fe (1 1 0).

**Fig. 10:**
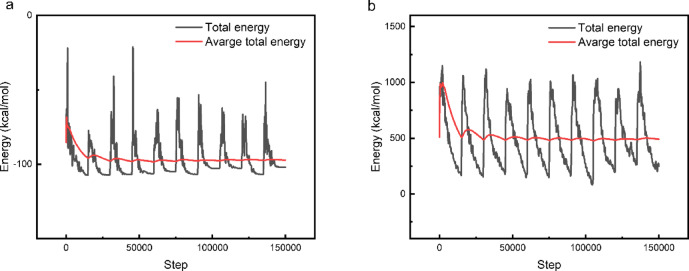
Energy profile of Q-Ar/Fe(110) annealing in gas (a) and simulated acid media (b).

*MC* simulations are a powerful tool for predicting and simulating the adsorption of Q-Ar onto Fe. The locating modules for adsorption available in MS.06 software can be utilized for these simulations. Figure 9 shows the most stable adsorption configurations of Q-Ar on the Fe(110) surface, as further supported by the energy profiles of the annealed systems in both gas **(**Fig. 10a**)** and simulated acidic media (Fig. 10b).The observed parallel and flat orientations of *Q-Ar* molecule suggest a strong likelihood of these compounds being adsorbed onto the *C-steel* surface, thereby reducing the susceptibility of the metal to corrosion^17,74^.The data in Table 6 demonstrated *E*_*ads*_ with higher negative values for *Q-Ar* on the Fe (110) surface^75,76^. The observed parallel and flat orientations of *Q-Ar* illustrate their strong spontaneous adsorption over the *C-steel* surface. The E_ads_ value obtained in simulated acidic solution phase was more than this obtained in vacuum phase. which suggests that the formation of hydrogen bonds among water molecules and the oxygen and nitrogen atoms of the Q-Ar facilitated a closer proximity of the Q-Ar to the Fe(110) surface^75,77^. This enhanced interaction between the Q-Ar and the metal surface improved the donor-acceptor interaction efficiency, ultimately suppressing the corrosion rate of the *C-steel*. Furthermore, the higher *E*_*ads*_ value for Q-Ar compared to water molecules, hydronium ions, and chloride ions indicate that the Q-Ar has a stronger adsorption affinity on the *C-steel* surface. This stronger affinity allows the Q-Ar to displace the aggressive ions adsorbed on the *C-steel* surface, effectively inhibiting corrosion as suggested by EIS data^73,78,79^.

The results obtained by *MCs* were matched with the parameters derived by *DFT*, and these findings collectively indicate the possibility of using the investigated quaternary ammonium salt compound (*Q-Ar*) as corrosion inhibitor for *C-steel*.

**Table 6:** The outputs energies calculated by MC simulation for *Q-Ar* in gas and simulated liquid phases on Fe (1 1 0).

Inh.		E_T_ (kJ/mol)	E_ads_ (kJ/mol)	E_rig_. (kJ/mol)	E_def_. (kJ/mol)	(dE_ads_/dNi) (kJ/mol)			
						Q-Ar	H_2_O	H_3_O^+^	Cl^−^
***Gas***		−180.7	−226.46	−178.316	−48.159	−226.468			
**Liquid**		−1270.02	−2329.54	−1315.86	−1013.68	−280.901	−34.678	−156.075	−140.783

### Surface analysis

**Fig. 11:**
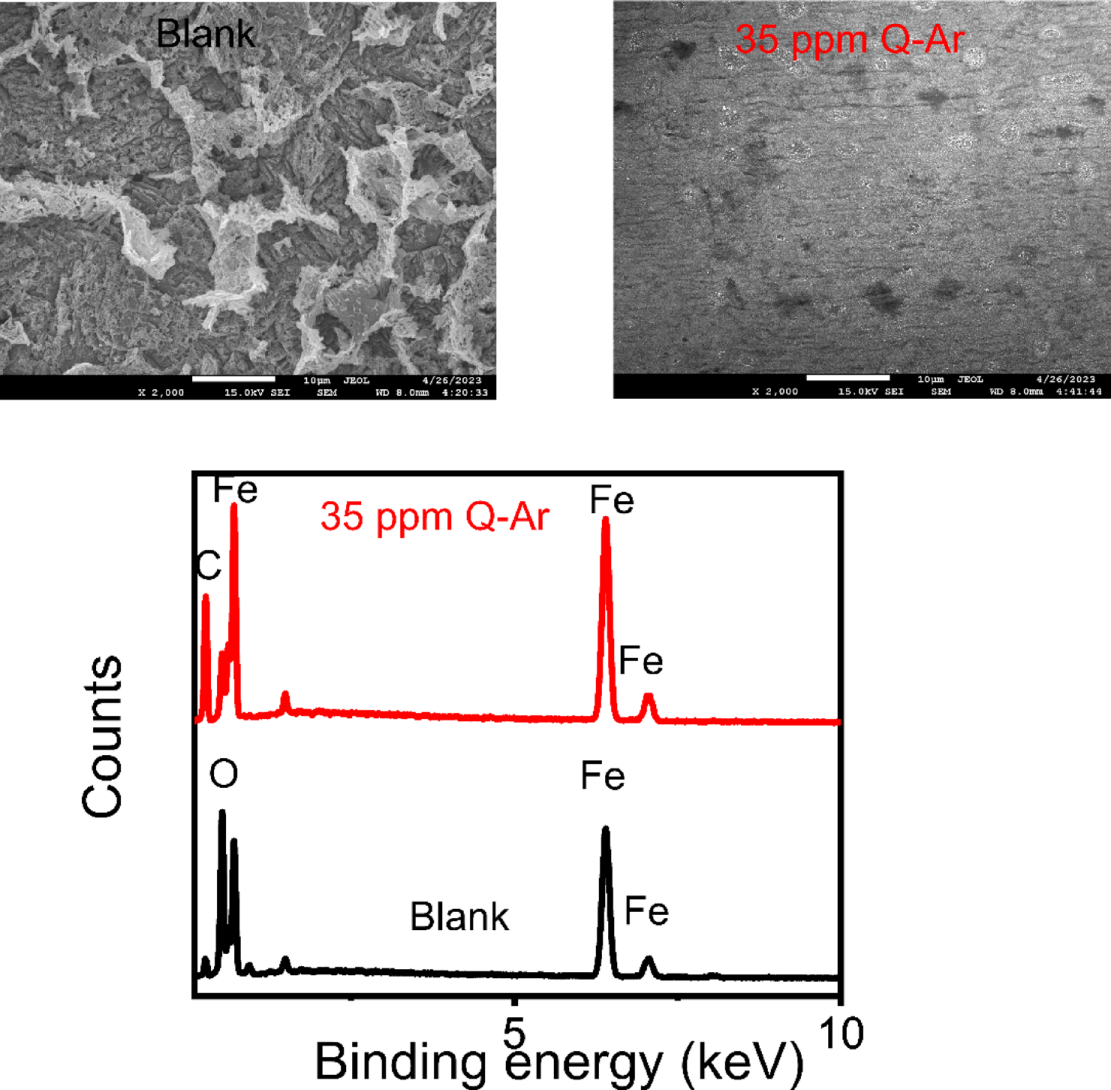
SEM and EDX for C-steel in 1 M HCl free and containing 35 ppm of Q-Ar after 6 h immersion.

*SEM* and *EDX* techniques were employed in conjunction with electrochemical measurements to examine the corrosive impact on 1 M HCl for 6 h without and with 35 ppm *Q-Ar*. In Fig. 11, *SEM* images show the metal surface suffered severe scratches and damage when exposed to HCl, resulting in the production of numerous corrosion by-products such as Fe-chloride and oxides. Unlike in the presence of the *Q-Ar* compound, the morphology of the metal surface showed an improvement due to protective film reaction by *Q-Ar* molecules acting as a shielding barrier separating the *C-steel* from the corrosive HCl. The *Q-Ar* film on the metal surface remained insoluble in 1 M HCl medium and was formed via strong chemical bonds between the *Q-Ar* compound and iron. This film effectively provides maximum insulation for the metal surface against the corrosive solution^47,80,81^. The percentage of oxides decreases from 19.79% to 7.36%, the Fe content decreases from 73.43% to 65.9% and the carbon content increases from 6.78% to 26.74% due to the adsorption of *Q-Ar* inhibitor, as shown in the *EDX* spectra (Fig. 11)^82^.

### Inhibition mechanism

**Fig. 12:**
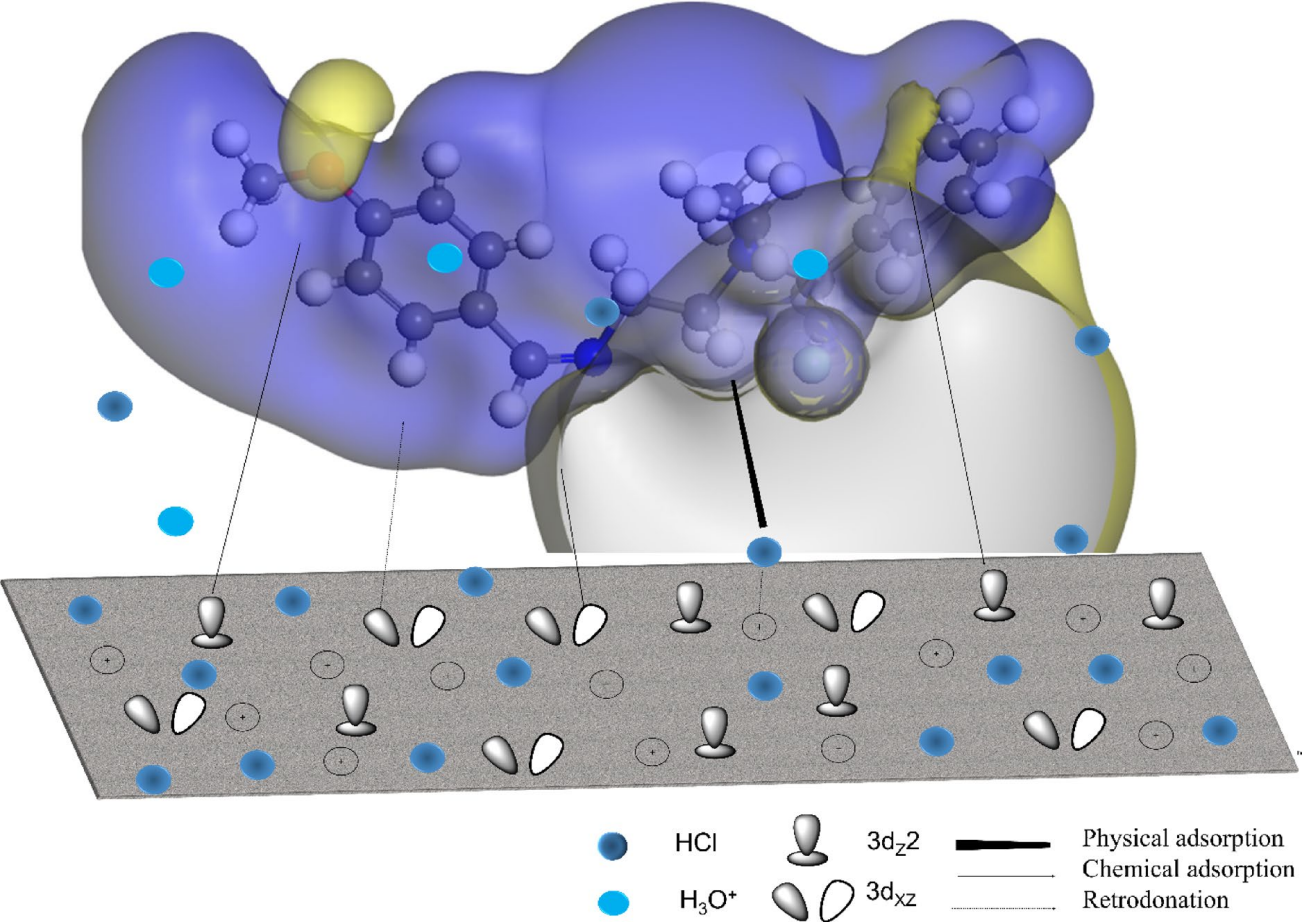
Suggested adsorption mechanism of *Q-Ar* over *C-steel* surface.

Figure 12 illustrates the proposed adsorption mechanism of Q-Ar molecules on the C-steel surface, highlighting the coexistence of both physical and chemical adsorption modes. In the first stage, physical adsorption occurs through electrostatic interactions between the positively charged quaternary nitrogen centers (–N⁺=) in the Q-Ar molecule and the negatively charged sites (Cl⁻ ions) adsorbed on the C-steel surface. This interaction facilitates the initial attachment of Q-Ar molecules onto the C-steel surface, forming an electrostatically stabilized layer. Additionally, chloride ions (counter ions) act as a bridge, linking the positively charged inhibitor centers with the partially negatively charged sites on the C-steel surface, thus enhancing surface coverage and uniformity.

15 $$\:{Q-Ar}^{+}.{Cl}^{-}+Fe\to\:Fe-({{Q-Ar}^{+}.{Cl}^{-})}_{ads}$$

Subsequently, chemical adsorption (chemisorption) becomes dominant through donor–acceptor interactions between the lone pair electrons of heteroatoms (N and O) and the π-electron systems (C = N and aromatic rings) in Q-Ar, and the partially vacant 3 d orbitals of surface iron atoms. This leads to the formation of strong coordination bonds, resulting in a compact and stable protective film that prevents the penetration of aggressive chloride ions and inhibits metal dissolution.


16


Furthermore, back-donation (retrodonation) from filled 3 d orbitals of iron to the antibonding orbitals (π*) of the Q-Ar molecules further strengthens the metal–inhibitor bond, enhancing the stability of the adsorbed layer (Q-Ar—Fe).


17


As a result, Q-Ar forms a mixed adsorption layer (physical + chemical) that reduces or blocked the available sites for iron ionization ($\:\mathrm{F}\mathrm{e}\ne\:{\mathrm{F}\mathrm{e}}^{++}$)and for proton reduction ($\:{\mathrm{H}}^{+}\ne\:{\mathrm{H}}_{2\uparrow\:}$).

## Conclusion

Newly synthesized quaternary ammonium salt (*Q-Ar*) based on Schiff-base with unique chemical structure was confirmed by *FTIR* and *¹HNMR* spectroscopy. Q-Ar investigated as corrosion inhibitor for *C-steel* in 1 M HCl under various conditions using electrochemical techniques. Potentiodynamic polarization (*PDP*) studies revealed their mixed inhibition performance for both anodic and cathodic *C-steel* reactions, indicating their ability to block active sites of the C-steel.This ends to dropping the i_corr_ of C-steel to 0.0346 mA/cm^2^ compaired to the blank counterpart value (0.5712 mA/cm^2^). The addition 35 ppm of Q-Ar decreased the double-layer capacitance of *C-steel* to 17.27 µF/cm^2^, while increasing the charge transfer resistance (*R*_*ct*_) to 737.3 Ω.cm^2^ confirming its impedance effects for C-steel corrosion reaction. *Q-Ar* demonstrated effective corrosion inhibition even under harsh conditions of temperature and immersion time, with only a slight decrease in inhibition efficiency (approximately 13%) observed. The adsorption of Q-Ar onto the *C-steel* surface follows a Langmuir adsorption isotherm, suggesting a chemical mechanism at room temperature. Thermodynamic activation studies revealed the energy barrier effect of *Q-Ar* on the *C-steel* corrosion reaction, where the activation energy of C-steel reaction was enhanced after Q-Ar addition. *MC* simulations further confirmed the effective adsorption affinity of Q-Ar compounds. Surface analysis studies confirmed the effectiveness of *Q*-Ar where the C-steel morphology was more smoother compared to the absence of Q-Ar. Overall, the unique molecular structure of Q-Ar—rich in electroactive centers and π-electron systems—facilitates strong adsorption and film formation on the steel surface, making it a highly effective and durable corrosion inhibitor for acidic environments.

## Supplementary Information

Below is the link to the electronic supplementary material.

Supplementary Material 1

## Data Availability

All data generated or analyzed during this study are included in this manuscript.
