# Supplementary material for: Corrosion inhibition mechanism of a functionalized schiff base–derived quaternary ammonium salt for carbon steel in 1 M HCl: electrochemical, adsorption, and theoretical studies
Source: Sci Rep. 2026 Apr 4;16:11618. doi: 10.1038/s41598-026-41236-5 (PMC13056983; doi:10.1038/s41598-026-41236-5)
Supplement: Supplementary file 1 — Supplementary Material 1 [file 41598_2026_41236_MOESM1_ESM.docx]

**Corrosion Inhibition Mechanism of a Functionalized Schiff Base–Derived Quaternary Ammonium Salt for Carbon Steel in 1 M HCl: Electrochemical, Adsorption, and Theoretical Studies**

Mohamed I. Ahmed^1^, M AbdEl-Raouf^1^, MA Migahed^1^, Magdy A.M. Ibrahim^2^, Sameh A. Rizk^2^, NM El Basiony^1^*

^1^ Egyptian Petroleum Research Institute, Nasr City 11727, Cairo, Egypt

^2^ Chemistry department, Faculty of Science, Ain Shams University, Cairo, Egypt


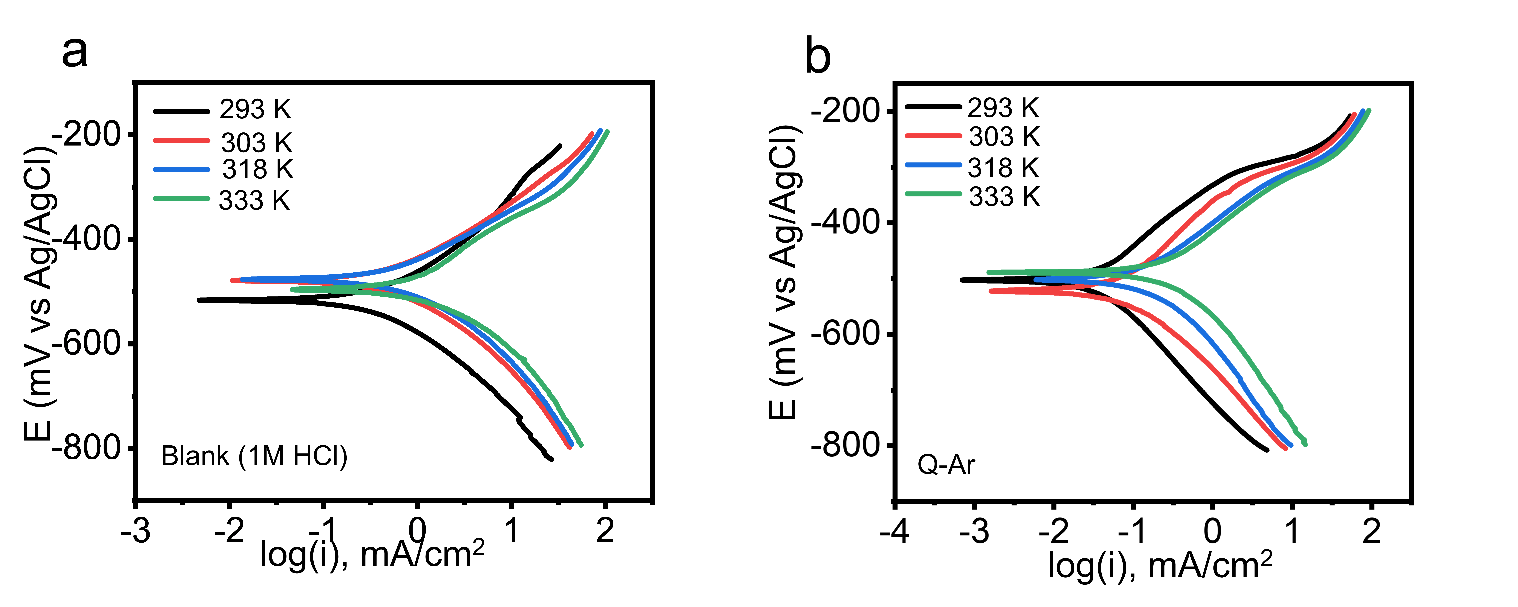


Fig.1s: Potentiodynamic polarization curves of C-steel steel in 1M HCl at different temperature in absence (a) and presence of 35 ppm Q-Ar (b).


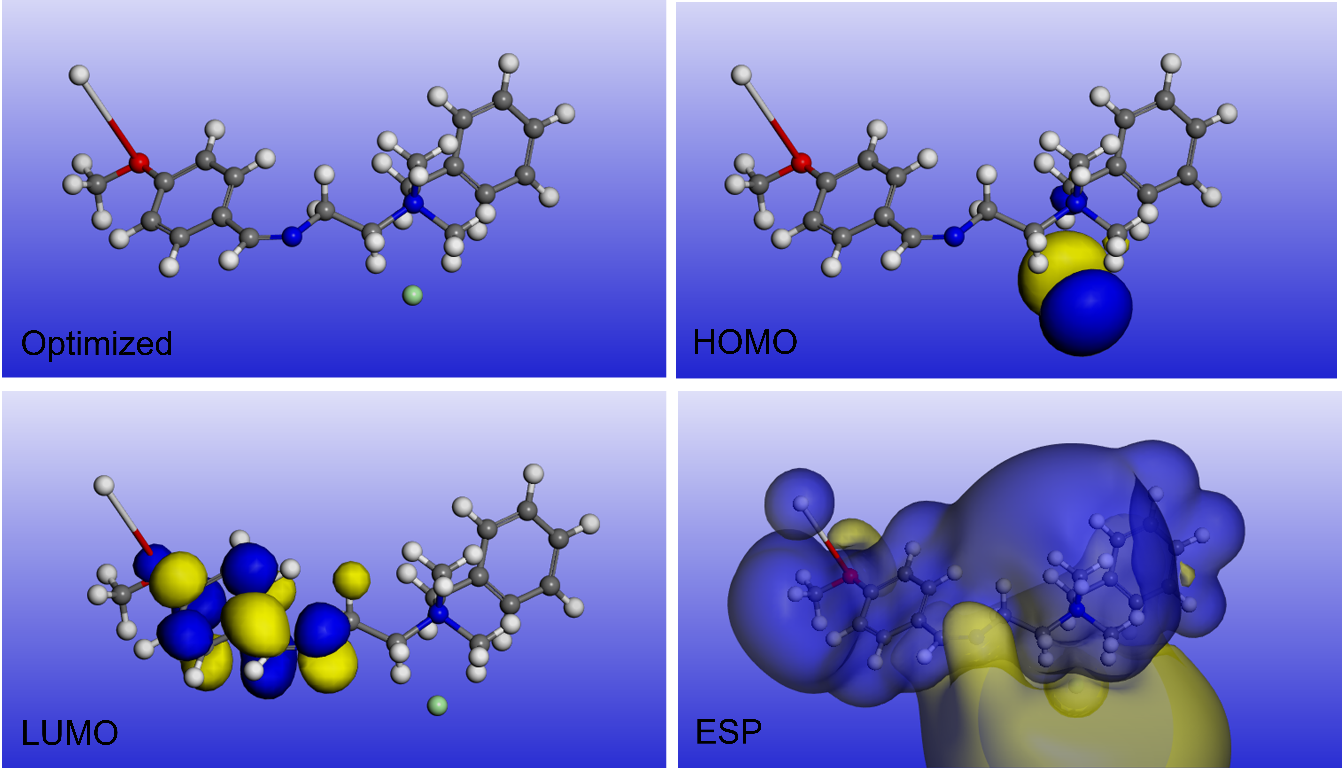


Fig.2s: Optimized geometry structure, HOMO, LUMO, and ESP of the protonated Q-Ar (Q-Ar-H^+^).

Table 1s: Tafel parameters of *C-steel* in absence and presence of 35 ppm from *Q-Ar* at different temperatures.

| ***Inh*.** | **Temp.** °C | ***E*_corr_,** (mV)  Vs. Ag/AgCl | **i_corr_** (mA/cm²) | ***β_a_*** (mV/dec) | ***-β_c_*** (mV/dec) | ***CR***  (mm/year) | **Ɵ** | ***IE*%** |
| --- | --- | --- | --- | --- | --- | --- | --- | --- |
| **Blank** | 20 | -516.3 | 0.5712 | 120.7 | 133.8 | 6.626385 | --- | --- |
|  | 30 | -478.8 | 1.2811 | 143.4 | 167.3 | 14.8618 | --- | --- |
|  | 45 | -476.5 | 1.5331 | 134.8 | 173.9 | 17.78521 | --- | --- |
|  | 60 | -495.9 | 1.8501 | 149.4 | 158.7 | 21.46266 | --- | **---** |
|  |  |  |  |  |  |  |  |  |
| ***Q-Ar*** | 20 | -502.6 | 0.0346 | 130.5 | 142.9 | 0.401388 | 0.93942 | 93.942 |
|  | 30 | -521.6 | 0.1415 | 190.6 | 162.7 | 1.641515 | 0.88954 | 88.954 |
|  | 45 | -501.4 | 0.2395 | 138.6 | 184.0 | 2.778395 | 0.84378 | 84.378 |
|  | 60 | -488.8 | 0.3310 | 131.5 | 160.3 | 3.839869 | 0.82109 | 82.109 |

Table 2s: *EIS* parameters for *C-steel* 1 M HCl in absence and presence of 35ppm from *Q-Ar* at different immersion time.

| ***Inh*.** | **t, min** | ***R*_s_,** (Ω.cm^2^) | ***R*_ct_** (Ω.cm^2^) | **Ɵ** | ***IE*%** |
| --- | --- | --- | --- | --- | --- |
|  |  |  |  |  |  |
| **Blank** | 30 | 3.16 | 46.2 | --- | --- |
|  | 60 | 2.02 | 49.87 | --- | --- |
|  | 120 | 2.58 | 62.34 | --- | --- |
|  | 240 | 2.45 | 64.53 | --- | --- |
|  | 360 | 2.49 | 74.08 | --- | --- |
| ***Q-Ar*** | 30 | 3.79 | 737.3 | 0.9373 | 93.73 |
|  | 60 | 3.77 | 582.1 | 0.9143 | 91.43 |
|  | 120 | 3.37 | 503.3 | 0.8761 | 87.61 |
|  | 240 | 4.04 | 488.5 | 0.8679 | 86.79 |
|  | 360 | 4.27 | 466.2 | 0.8312 | 83.12 |

Table 3s: Comparison between the inhibition efficiency of the prepared inhibitor *Q-Ar* and other investigated inhibitors for *C-Steel* in 1 M HCl.

| **Compound** | **concentration** | ***IE*%** | | **Reference** |
| --- | --- | --- | --- | --- |
|  |  | **PDP** | **EIS** |  |
| **PMAA2** | 800 ppm | 89.34 | 84.96 | [1] |
| **PMAA3** |  | 91.45 | 90.05 |  |
| **Di-Sb** | 1×10^-3^ M | 92.95 | 93.02 | [2] |
| LNEt | 1×10-4 M | 79.06 | 75.48 | [3] |
| **PSB7** | 1×10^-3^ M | 94 | 92.7 | [4] |
| **PSB8** |  | 93 | 94.3 |  |
| **Q235** | 250 ppm | 92.2 | 76.0 | [5] |
| ***Q-Ar*** | 35 ppm (1.18×10^-4^ M) | 93.94 | 93.73 | Present study |

References

[1] X. Li, J. He, B. Xie, Y. He, C. Lai, W. Wang, J. Zeng, B. Yao, W. Zhao, T. Long, 1, 4-Phenylenediamine-based Schiff bases as eco-friendly and efficient corrosion inhibitors for mild steel in HCl medium: experimental and theoretical approaches, J. Electroanal. Chem. 955 (2024) 118052.

[2] A. Elaraby, K. Faisal, S.K. Mohamed, E.A. El-sharkawy, Multi-scale quantum ( DFT , MCs and MDs ) insights and electrochemical validation of di-imine Schiff base inhibitor for carbon steel corrosion control in 1 M HCl solution, 42 (2025) 102615.

[3] H. Debab, I. Selatnia, H. Hamani, T. Douadi, M. Messali, S. Benabid, N. El Messaoudi, M.M. Abou-krisha, A.A. Alrashdi, H. Lgaz, Interfacial Mechanisms and Performance of a Diimine Schiff Base Inhibitor for Carbon Steel in 1 M HCl: Advanced Modelling Coupled with Experimental Characterization, Mater. Today Commun. (2025) 114470.

[4] A. Lahhit, C. Zannagui, I. Azghay, A. Elyoussfi, Y. Ouzidan, M. El Massaoudi, F. Mourabit, M. Ahari, H. Amhamdi, S. El Barkany, Investigating the efficacy of phenolic Schiff bases as corrosion inhibitors for mild steel in acidic environments: Integrating experimental and theoretical approaches (Part B), J. Mol. Struct. 1318 (2024) 139369.

[5] M.M.Y. Modwi, H. Feng, M.K. Hadi, N. Chen, J. Hou, E. Kamal, K. Yang, Eco-friendly corrosion inhibitor of Q235 carbon steel in 1.0 M HCl by Isatin/Chitosan Schiff base, J. Mol. Struct. 1321 (2025) 139592.
